# Genetic Diversity and Population Structure Analysis in Guar

**DOI:** 10.3390/plants13223183

**Published:** 2024-11-13

**Authors:** Shubham Malani, Waltram Ravelombola, Aurora Manley, Hanh Pham

**Affiliations:** 1 Texas A&M AgriLife Research, 11708 Highway 70 South, Vernon, TX 76384, USA; 2 Soil and Crop Sciences, Texas A&M University, 370 Olsen Blvd., College Station, TX 77843, USA; 3 Texas A&M AgriLife Research, 1102 East Drew Street, Lubbock, TX 79403, USA

**Keywords:** guar, genetic diversity, population structure, gene pool

## Abstract

Guar [*Cyamopsis tetragonoloba* (L.) Taub] was domesticated in India and Pakistan. It is mainly self-pollinated, bushy, and deeply tap-rooted. Guar seed endosperm contains galactomannan gum, which is used in many food products, pharmaceuticals, cosmetics, explosives, meat products, and pet foods, and in the textile industry, yet its genetic diversity remains largely underexplored. Using 7000 high-quality single nucleotide polymorphism (SNP) markers acquired from genotyping by sequencing (GBS), we analyzed the genetic diversity and population structure in 225 guar accessions from India, Pakistan, and the United States. Structure Harvester revealed that K = 3 had the best delta K, whereas K = 2 had the second-highest delta K. Three major genetic clusters (K = 3) were identified using population structure analysis, utilizing an admixture model: 156 accessions (69.3%) were classified into Q1, 23 accessions (10.2%) in Q2, and 16 accessions (7.1%) in Q3. The remaining 30 accessions (13.3%) were included in the admixture. In all three of the subpopulations at K = 3, most of the guar accessions came from India. We also found that these clusters mostly correlated with geographic origins. Results showed that the Q2 and Q3 subpopulations included only guar accessions from India. Genetic resources from Q2 and Q3 may represent an untapped reservoir for introducing beneficial variety into the U.S. breeding populations. This genetic diversity and population structure analysis of the guar gene pool will be of interest to conduct allele mining and donor parent selection for the development of new and better guar germplasm for desired traits.

## 1. Introduction

Guar [*Cyamopsis tetragonoloba* (L.) Taub], also known as cluster bean, is a diploid (2n = 2x = 14) annual legume crop grown in many parts of the world with high temperature and low precipitation. Guar was domesticated in India and Pakistan [1]. Guar varieties that are currently cultivated in India include HG-884, RGC-1031, and GAUG-13 [1]. Examples of guar varieties grown in Pakistan include BR-21 and BR99 [1]. *Cyamopsis senegalensis*, an African species, is thought to be the source from which the cultivated species was domesticated [2]. Around the world, guar is cultivated in many semi-arid areas including Sudan, the Mediterranean region, the United States, Australia, Pakistan, India, and other places [3,4]. The guar plant is a crop that is mainly self-pollinated, bushy, and tap-rooted deeply [5,6].

Guar seed endosperm produces galactomannan gum, which is a polysaccharide working as a binding agent and gel when mixed with water that gives perfect viscosity and thickness [7]. It is used in many food products, pharmaceuticals, cosmetics, explosives, meat products, and pet foods, and in the textile industry [5,8]. The guar gum used by the oil and gas sector as a lubricant during the hydraulic fracturing process has significantly contributed to the recent surge in guar usage [4]. Only nine varieties have been released in the United States in last 60 years, which requires the need for guar germplasm improvement [9,10]. The United States has the largest guar gum market, evaluated at $1.1 billion [1]. In addition, guar can be also used as a green manure or rotational crop. It can fix atmospheric nitrogen via association with rhizobacteria [1], which improves soil fertility. Immature guar pods can be consumed as vegetables.

To address global issues affecting food security, sustainability, and climate change adaptation, plant breeders utilize the diversity of genetic resources to produce new and improved crop cultivars [11]. The fundamental component of biodiversity and variety within and between species, as well as within ecosystems, is genetic diversity. Natural genetic diversity within crop species has been utilized since the beginning of agriculture to fulfill subsistence food requirements [12]. Understanding the genetic linkages and diversity of germplasm among breeding materials is important to develop crop improvement strategies [13].

There has historically been little focus on genetic improvement and very little breeding effort done to increase guar yield. It is essential to understand the level of genetic diversity of crops and use this information for crop improvements [14]. Gresta et al. [15] evaluated the genetic diversity of eight guar accessions from South Africa, India, and the United States using amplified fragment length polymorphism (AFLP) markers. Eight primer pairs were used to successfully amp up the AFLP markers, and the findings indicated that the primer pairs were effective. Gresta et al. [15] reported a correlation between the country of origin and the grouping of the guar accessions using molecular markers.

The introduction of high-throughput technology has revolutionized the use of single nucleotide polymorphisms (SNPs) as molecular markers [16]. It is easier to assess genetic variation and examine patterns of variation across markers and between populations using SNPs because they are binary markers [17]. SNP genotyping can help with breeding efforts for guar by fine-mapping relevant genes. Using genomic or marker-assisted selection, molecular markers may be used to conduct guar molecular breeding. In addition, single nucleotide polymorphisms are useful markers for population structure analysis, high-resolution genetic map creation, and the identification of marker–trait correlations in association mapping studies [18]. Because of their richness, extensive genomic coverage, availability of neutral variation and specific loci, quick and high-yield genotyping, and low error rate, SNP molecular markers have been extensively utilized to research genetic diversity [19]. However, the use of SNP markers to assess the genetic diversity of guar remains limited. Therefore, the objective of this study is to assess the genetic diversity and to conduct a population structure analysis in guar using SNP data.

## 2. Results

### 2.1. Single Nucleotide Polymorphism Profiling

A total of 64,236 raw single nucleotide polymorphisms (SNPs) were obtained from genotyping by sequencing (GBS). After discarding the SNPs with more than 15% heterozygous calls, 15% missing data, and those with minor allele frequency less than 5%, a total of 19,007 high-quality SNPs were retained. A total of 7000 randomly selected SNPs (~1000 SNPs per chromosome) from the high-quality SNP set were used to conduct population structure and genetic diversity analysis. The six SNP types that were found within the selected 7000 SNPs were [AG] 1496 (21.4%), [CT] 1401 (20.0%), [GT] 1237 (17.7%), [AT] 1120 (16.0%), [AC] 901 (12.9%), and [CG] 845 (12.1%).

### 2.2. Population Structure and Genetic Diversity

**Figure 1:**
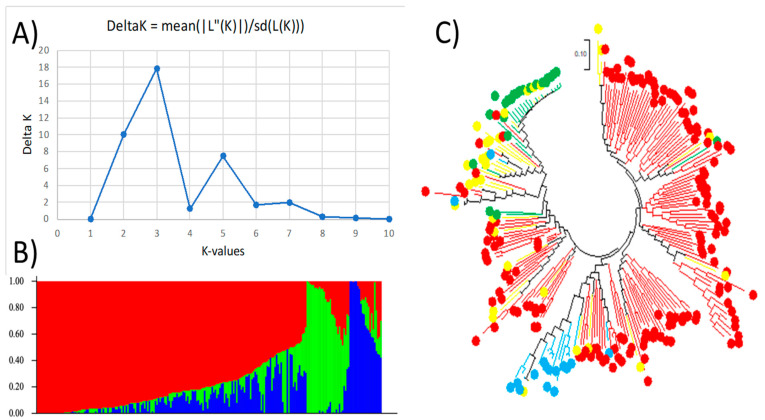
Genetic diversity and population structure at K = 3. (**A**) Delta K values determined by Structure Harvester for various numbers of populations assumed (K) in STRUCTURE analysis. (**B**) Population structure of 225 guar accessions divided into 3 subpopulations using STRUCTURE 2.3.4, where each accession is shown on the x-axis and the subgroup membership is displayed on the y-axis. The color coding (Q1: red, Q2: green, Q3: blue) indicates the dispersion of the accessions to distinct subpopulations. (**C**) Maximum likelihood (ML) phylogenetic tree of the 225 guar accessions drawn using MEGA 7. The color codes are consistent in (**A**,**B**). Admixture is shown in yellow.

**Figure 2:**
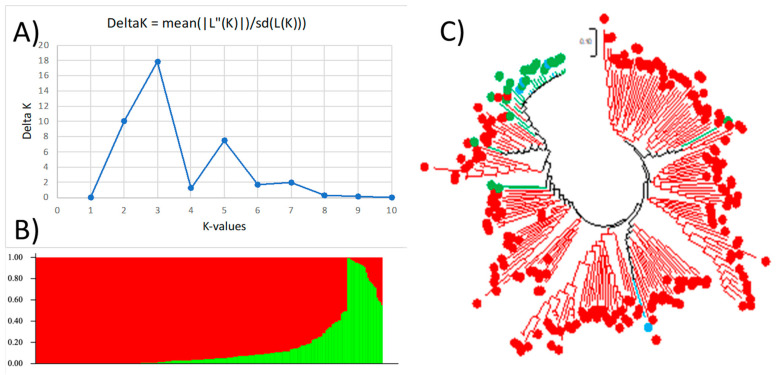
Genetic diversity and population structure at K = 2. (**A**) Delta K values determined by Structure Harvester for various numbers of populations assumed (K) in STRUCTURE analysis. (**B**) Population structure of 225 guar accessions divided into 2 subpopulations using STRUCTURE 2.3.4, where each accession is shown on the x-axis and the subgroup membership is displayed on the y-axis. The color coding (Q1: red, Q2: green) indicates the dispersion of the accessions to distinct subpopulations. (**C**) Maximum likelihood (ML) phylogenetic tree of the 225 guar accessions drawn using MEGA 7. The color codes are consistent in (**A**,**B**). Admixture is shown in blue.

Structure Harvester showed an optimal delta K for K = 3, indicating that this guar panel has three subpopulations (Q1, Q2, and Q3) (Figure 1. A second delta K peak was also found for K = 2, showing that this population could also be divided into two subpopulations (Q1 and Q2) (Figure 2). For K = 3, 225 accessions were assigned to one of the three subpopulations using a 0.55 probability threshold, with 156 accessions (69.3%) being grouped into Q1, 23 accessions (10.2%) in Q2, and 16 accessions (7.1%) in Q3. The admixture contained the remaining 30 out of 225 accessions (13.3%) (Figure 1) (Table S1). With respect to K = 2, Q1 consisted of 198 accessions (88%), followed by Q2 with 23 accessions (10.2%), and 4 (1.8%) as admixture.

The maximum likelihood (ML) approach was used to create the phylogenetic trees using MEGA 7. Figure 1 shows the genetic diversity combined with the population structure analysis at K = 3. The first cluster, Q1, is shown in red, the second cluster, Q2, in green, and the third cluster, Q3, in blue (Figure 1B). The phylogenetic trees are colored using the same palette, with red circles denoting accessions from Q1, green indicating those from Q2, blue showing the Q3 cluster, and yellow for the admixture (Figure 1C). The results showed that more than 90% of the genotypes within Q2 and Q3 are grouped together on the phylogenetic tree, indicating consistent results between the genetic diversity and population structure for these two subpopulations. The first subpopulation, Q1, was grouped into two categories based on the genetic diversity analysis. This indicates that genetic diversity analysis can further investigate the level of diversity within this subpopulation. As expected, the admixture was scattered within the phylogenetic tree.

Figure 2 shows the genetic diversity combined with the population structure analysis at K = 2. The first cluster, Q1, is shown in red, the second cluster, Q2, in green, and the admixture in blue (Figure 2B). A correlation between genetic diversity and population structure is found because the two subpopulations are majorly grouped into two categories, which you can see as red being the largest and green being the second largest group (Figure 2B). The phylogenetic tree (Figure 2C) shows that the accessions under each cluster tend to be grouped together, indicating that the structural analysis results are congruent with the genetic diversity results.

### 2.3. Population Structure by Country of Genotype Origin

Genetic diversity was further examined by country of genotype origin. Table 1 and Table 2 show that the proportion of accessions for each subpopulation varied among the three countries of origin of the guar genotypes (India, Pakistan, and the U.S.). At K = 3, most of the guar accessions were from India within each of the three subpopulations. In addition, no accessions from Pakistan or the U.S. were found within the Q2 and Q3 subpopulations, indicating that these two subpopulations are specific to guar accessions from India. The Indian accession consisted of a Q1 cluster (56.44%), admixture (12.98%), Q2 cluster (10.22%), and Q3 cluster (7.11%). For the Q1 cluster, the U.S. and the Pakistan clusters, respectively, represent 5.33% and 6.67% of the guar accessions for that subpopulation. These results were also consistent at K = 2, where guar accessions from Pakistan and the U.S. were not found in the second subpopulation, Q2. The Q1 cluster was the most prevalent in the Indian population (74.67%), followed by the Q2 cluster (10.22%), with admixture accounting for just 1.78% of the studied accessions.

Table 3 shows the number of effective alleles per locus (Ne), the unbiased Nei genetic diversity (uh), and the percentage of polymorphic loci (%P). Ne varied from 1.51 to 3.49, with chromosome 1 having the highest Ne value. The uh value ranged from 0.341 to 0.624, with chromosome 1 having the highest uh value. The percentage of polymorphic loci varied from 51.8 to 71.2%, with chromosome 1 having the highest %P value.

## 3. Discussion

Our comprehensive analysis of 225 guar accessions demonstrated a robust population structure that split the guar germplasm into three primary subclusters and a modest level of genetic diversity. The conservation and breeding of guars will be significantly impacted by these discoveries. These results highlight the higher level of genetic diversity present in Indian guar germplasm as opposed to the U.S. and Pakistan; the Indian gene pool is composed of many ancestral groups. The Q1 cluster dominance indicates that this ancestral group has contributed the most genetic material to the current guar accessions. As mentioned above, Q2 and Q3 clusters consisted of only genotypes from India, indicating the existence of genetic variation specific to a subpopulation exclusive to the Indian accessions, which differs from the common ancestral diversity of all locations, as shown by Q1. To completely understand the origins of the population substructure on the Indian subcontinent, more research on subpopulation differentiation and admixture events across India’s complicated demographic history is needed. The various accession percentages of each group within the three clusters demonstrated the existence of a regional or geographic component influencing the genetic diversity and population structure of guar. These results also demonstrate that the countries of origin of guar accessions can affect population structure.

Using SNP markers, several prior research studies have investigated genetic diversity and population structure in various crops such as legumes like chickpea [20], soybean [21], and pigeon pea [22] and cereals like rice [23] and sorghum [24]. These studies often show regional trends in population structure, with accession groupings depending on country or place of origin, similar to our findings in guar. They also show that crop gene pools still contain a moderate amount of genetic variation even though there might be less genetic diversity because of their domestication in specific regions. The patterns of genetic diversity found in a crop species can be influenced by its breeding strategy. Due to inbreeding, guar, an annual self-pollinating plant, undergoes genetic bottlenecks every generation [25]. Outcrossing species, on the other hand, can maintain higher levels of variety over generations because they have higher rates of gene flow and recombination between individuals [12].

According to these findings, guar accessions typically cluster together in groupings that correlate with their geographical origins in Pakistan, India, and the U.S. Most of them are clustered in India because of their earlier domestication (Table 1 and Table 2). A large number of Indian accessions came from cultivated types created by Indian breeding programs with a strategic focus. Such breeding would strengthen and concentrate particular genomic areas over others through recurrent crossing, selection, and population enrichment for features preferred by agronomy. Shown in Q2 and Q3, this human-driven genome shaping might appear as a unique genetic cluster. This shows that population structure and genetic diversity are highly correlated with the country of origin, most likely as a result of selection affecting locally and of founder effects during introduction to new places. According to Kumara et al. [14], most genotypes from different origins are clustered into the same cluster in guar, although some individuals fall into separate clusters.

Genetic diversity is used by breeders to create novel cultivars that have better agronomics, such as increased yield and resistance to both biotic and abiotic stress, as well as to enhance the nutritional value of food for the world’s expanding population [11]. Crop genetic diversity analysis in space and time is needed to attain sustainable crop production [26]. With the use of genome sequence data, it is now possible to describe genetic diversity in a wide variety of plant germplasm, including contemporary cultivars, landraces, wild and weedy ancestors, and breeding materials. Breeders may monitor, assist in identifying, and select for valuable variability by using this sequence language, leading to the development of new and better varieties [27]. In addition, genetic diversity and analysis can aid in better understanding complex crop traits. For example, Thakur and Randhawa [28] studied the different molecular markers, including SSR, SNP, and InDel, to understand the molecular mechanism of root development, gum synthesis, and stress tolerance. Grigoreva et al. [29] discovered the development of an SNP set that enables the identification of reliable genetic markers of significant agrobiological traits in guar.

The clear description of the population substructure reveals diversity reservoirs that are divided across different germplasm groupings. Through targeted introgression and intercrossing, we may strategically utilize inter-subpopulation variety by comprehending these patterns of genetic divergence. In breeding populations, the rate of genetic gain for complex polygenic characteristics may be accelerated by combining advantageous alleles distributed throughout subpopulations. Therefore, the reported SNP data used for population structure and genetic diversity analysis in this study will be critical in designing effective guar breeding strategies such as parent selection and germplasm enhancement through molecular breeding.

## 4. Materials and Method

### 4.1. Plant Materials

A total of 225 guar genotypes were used to evaluate genetic diversity and population structure. The 225 guar genotypes consisted of 222 USDA accessions and 3 commercial cultivars (‘Kinman’, ‘Lewis’, and ‘Santa Cruz’) from the U.S. The guar genotypes were acquired from the Griffin, Georgia, USA-based Plant Genetic Resources Conservation Unit. These genotypes were originally from different countries, including India, Pakistan, and the United States.

### 4.2. DNA Extraction, Library Preparation, and Genotyping by Sequencing (GBS)

Genomic DNA was extracted from young, fresh guar leaves. After being kept at −80 °C for one night, the leaves were dried in a Lyophilizer^®^ (Salt Lake City, UT, USA). Then, guar leaves were ground using a Mixer Mill MM 400^®^ (Haan, Germany). DNA was extracted using the CTAB (hexadecyltrimethyl ammonium bromide) procedure as described by Kisha et al. [30]. After adding the DNA extraction buffer to every sample, the samples were centrifuged for 10 min at 13,000 rpm. The aqueous solution was then transferred into 2 mL tubes. A solution of 1 mL of chloroform–isoamyl alcohol (24:1) was added to each sample to remove proteins. After five minutes of centrifuging the samples at 10,000 rpm, the liquid supernatant was transferred to 2 mL tubes. The addition of 1 mL of isopropanol to every tube contributed to DNA precipitation in the mixture. The samples were then kept at −20 °C for the whole night. Following a 70 and 90% ethanol wash, the DNA pellets were dried for 30 min. To solubilize the DNA, 200 µL of 0.1× TE was added to each tube. Then, RNAse (3 µL) was supplied to each tube to remove RNA.

A NanoDrop 200c spectrophotometer (Thermo Scientific, Wilmington, DE, USA) was used to quantify DNA. DNA quality was checked using a 1% agarose gel stained with ethidium bromide gel dye. DNA sequencing was conducted using genotyping by sequencing (GBS) [31,32]. Sequencing was performed at Novogene (https://www.novogene.com/us-en/ accessed on 13 February 2024) using Illumina HiSeq series. For the DNA sequencing process, sticky end fragments were produced during the digestion of DNA by the restriction enzyme *ApeKI*. The restriction segments were ligated to two adapters consisting of P1 and P2. A 4–8 bp barcode and a forward Illumina Sequencing primer made up the P1 adapter or barcode. Reverse Illumina Sequencing primer was present in P2 adapters or common adapters. Every fragment had its 3′ end repaired and adenylated. The two primer pairs (forward and reverse Illumina Sequencing primers) were used for in situ polymerase chain reaction (PCR). On the other hand, DNA fragments may ligate to common/common, barcode/barcode, or barcode/common adapters. Same-ended samples could not be sequenced on the Illumina platform, so no reads were obtained. Sequencing was limited to fragments bearing both a barcode and a common adapter on both ends. A quality assessment of the GBS libraries was done before sequencing using Novogene’s procedure.

### 4.3. SNP Assembly, Mapping, Discovery, and Filtering

SNP assembly, mapping, and discovery were conducted using the Genome Analysis Toolkit (GATK) pipeline (https://gatk.broadinstitute.org/hc/en-us accessed on 13 February 2024). The reads were aligned using an in-house draft genome. GATK was also used to make the initial SNP calls. Guar genotypes with more than 15% of the SNP data missing were excluded from the study. SNPs with heterozygous calls of more than 15% and a minor allele frequency less than 5% were discarded from the study. Additionally, SNPs with more than 15% of missing data were also discarded. After SNP filtering, 19,007 SNPs predicted from GBS were retained.

### 4.4. Population Structure Analysis

The population structure of the guar accession panel was assessed using STRUCTURE 2.3.4 [33]. To evaluate the population structure (K), an admixture model and a correlated allele frequency model that were independent for each run were included in the study. Ten runs of each estimated K value were conducted. The burn-in period Markov Chain Monte Carlo (MCMC) length was 20,000. The number of MCMC iterations was set to 20,000 after the burn-in phase. Using Structure Harvester [34]; http://taylor0.biology.ucla.edu/structureHarvester/ (accessed on 13 February 2024), values of delta K and optimum K were calculated based on the method created by Evanno et al. [35]. Following the identification of the K optimum, a Q matrix containing the K vectors was created. Each guar genotype was assigned to a specific cluster (Q). The threshold probability to assign a guar genotype to a Q-group was set to 0.55. Using the “Sort by Q” option and the “STRUCTURE PLOT” option in STRUCTURE 2.3.4 [33], the bar plots were designed. The number of effective alleles (Ne), the unbiased Nei genetic diversity (uh), and the percentage of polymorphic loci (%P) were estimated using GenAlex v. 6.5 [36].

### 4.5. Population Diversity Analysis

MEGA 7 was used for phylogenetic tree building and genetic diversity analysis [36]. The statistical approach used was the maximum likelihood tree with the parameters determined by Shi et al. [37]. When creating the phylogenetic trees in MEGA 7, the population structure and the outputs including the Q clusters were imported. This allowed for a combined examination of genetic diversity. The “branch line” and “node/subtree marker” shapes for the subtrees for each cluster (Q) were colored in a manner consistent with the bar plots that STRUCTURE PLOTS exhibited.

## 5. Conclusions

In conclusion, patterns of genetic diversity and population structure in a set of 225 guar accessions from Pakistan, India, and the United States were described in this study. High polymorphism was found in the examined guar germplasm due to the abundance and high genome coverage of these markers, making them an exceptional and useful tool for screening the genome-wide genetic diversity of guar. The majority of accessions formed a main cluster (Q1), which was highly correlated with an Indian geographic origin, according to the population structure analysis, while all of the accessions from the U.S. and Pakistan fell under the Q1 cluster. The genetic uniqueness of Q1 relative to other subgroups such as Q2 and Q3 suggests the possibility of new allelic diversity in those other clusters that is not seen in the Q1 gene pool of the Pakistani and U.S. populations. In order to introduce favorable diversity into U.S. breeding populations, genetic resources from Q2 and Q3 may constitute an untapped pool. A sizable amount of the overall genetic diversity found was concentrated in this cluster, suggesting that a significant portion of the genetic variety found in cultivated guar germplasm is found in India. These findings hold significant value for attempts to conserve germplasm and for guar breeding projects. It might be useful to investigate the genetic diversity of guar before choosing parents used for future crossings and breeding efforts.

## Figures and Tables

**Table 1:** Population structure by country at K = 3.

	India	U.S.	Pakistan
Q1	56.44%	5.33%	6.67%
Q2	10.22%	0%	0%
Q3	7.11%	0%	0%
Admixture	12.89%	0%	0%

**Table 2:** Population structure by country at K = 2.

	India	U.S.	Pakistan
Q1	74.67%	5.33%	6.67%
Q2	10.22%	0%	0%
Admixture	1.78%	0%	0%

**Table 3:** Genetic diversity level of guar genotypes.

Chromosome	Ne	uh	%P
1	3.49	0.624	71.2
2	2.32	0.587	65.4
3	1.52	0.394	60.2
4	2.48	0.434	57.3
5	2.47	0.586	58.8
6	1.51	0.341	51.8
7	3.45	0.683	68.3

Note: Ne = number of effective alleles per locus, uh = unbiased Nei diversity, %P = polymorphism.

## Data Availability

Data are contained within the article.

## Supplementary Materials

The following supporting information can be downloaded at: https://www.mdpi.com/article/10.3390/plants13223183/s1, Table S1. Population structure matrix at K = 3. Table S2. Population structure matrix at K = 2.
