# Supplementary material for: Genetic Diversity and Population Structure Analysis in Guar
Source: Plants (Basel). 2024 Nov 13;13(22):3183. doi: 10.3390/plants13223183 (PMC11597707; doi:10.3390/plants13223183)
Supplement: Supplementary file 1 [file plants-13-03183-s001.zip › plants-3202304-supplementary.pdf]

Supplementary Table S1. Population structure matrix at K = 3.

Genotypes	Origin	Q1	Q2	Q3	Group
PI275322	India	0.938	0.061	0.001	Q1
PI288389	India	0.001	0	0.999	Q3
PI288409	India	0.509	0.004	0.488	Q1Q2Q3
PI288743	India	0.4	0.595	0.006	Q2
PI288754	India	0.342	0.625	0.033	Q2
PI288759	India	0.633	0.34	0.026	Q1
PI323002	India	0.509	0.486	0.005	Q1Q2Q3
PI338811	India	0.999	0	0.001	Q1
PI338865	India	0.994	0.002	0.004	Q1
PI340513	India	0.806	0.172	0.022	Q1
PI426634	Pakistan	0.817	0.076	0.107	Q1
PI158119	India	0.688	0.051	0.261	Q1
PI164765	India	0.967	0.012	0.021	Q1
PI253184	USA	0.881	0.05	0.069	Q1
PI271544	India	0.996	0.003	0.001	Q1
PI271548	India	0.952	0.046	0.002	Q1
PI288384	India	0.22	0.302	0.478	Q1Q2Q3
PI288431	India	0.337	0.589	0.074	Q2
PI288748	India	0.017	0.981	0.001	Q2
PI288752	India	0.134	0.864	0.002	Q2
PI288761	India	0.525	0.469	0.006	Q1Q2Q3
PI340514	India	0.821	0.076	0.103	Q1
PI593048	USA	0.772	0.079	0.149	Q1
PI593058	USA	0.837	0.039	0.124	Q1
PI671848	USA	0.825	0.012	0.164	Q1
PI116034	India	0.993	0.005	0.002	Q1
PI158120	India	0.955	0.044	0.001	Q1
PI158123	India	0.998	0	0.001	Q1
PI158126	India	0.888	0.109	0.003	Q1
PI163103	India	0.969	0.03	0.001	Q1
PI164170	India	0.96	0.001	0.039	Q1
PI164386	India	0.866	0.005	0.13	Q1
PI164477	India	0.901	0.002	0.098	Q1
PI164799	India	0.879	0.001	0.12	Q1
PI165511	India	0.945	0.026	0.029	Q1
PI176375	India	0.905	0.056	0.04	Q1
PI179682	India	0.871	0.046	0.083	Q1
PI179686	India	0.763	0.007	0.23	Q1
PI179928	India	0.998	0.002	0	Q1
PI180285	India	0.952	0.038	0.01	Q1
PI180433	India	0.834	0.024	0.142	Q1
PI182968	India	0.132	0.782	0.086	Q2
PI186477	India	0.818	0.089	0.093	Q1
PI198297	India	0.545	0.007	0.448	Q1Q2Q3
PI212988	India	0.937	0.003	0.061	Q1
PI214319	India	0.964	0.036	0.001	Q1

PI215591	India	0.998	0.001	0.001	Q1
PI217924	India	0.897	0.005	0.098	Q1
PI223685	India	0.743	0.012	0.246	Q1
PI236479	India	0.664	0.02	0.316	Q1
PI250214	Pakistan	0.999	0	0	Q1
PI250358	Pakistan	0.995	0.002	0.003	Q1
PI250360	Pakistan	0.996	0	0.004	Q1
PI253183	USA	0.915	0.001	0.084	Q1
PI253186	USA	0.921	0.001	0.079	Q1
PI254367	India	0.7	0.015	0.285	Q1
PI255928	India	0.703	0.016	0.281	Q1
PI262152	Pakistan	0.951	0.003	0.046	Q1
PI262154	Pakistan	0.897	0.099	0.004	Q1
PI262157	Pakistan	0.749	0.096	0.155	Q1
PI263874	India	0.722	0.098	0.181	Q1
PI263876	India	0.823	0.105	0.072	Q1
PI263879	India	0.754	0.19	0.056	Q1
PI263881	India	0.573	0.158	0.269	Q1
PI263883	India	0.237	0.179	0.585	Q3
PI263885	India	0.587	0.061	0.353	Q1
PI263888	India	0.65	0.115	0.235	Q1
PI263891	India	0.979	0.008	0.012	Q1
PI263893	India	0.979	0.019	0.002	Q1
PI263895	India	0.84	0.065	0.095	Q1
PI263897	India	0.909	0.022	0.068	Q1
PI263899	India	0.536	0.088	0.377	Q1Q2Q3
PI263901	India	0.697	0.191	0.112	Q1
PI268229	USA	0.999	0.001	0	Q1
PI271535	India	0.867	0.064	0.069	Q1
PI271541	India	0.962	0.038	0.001	Q1
PI271550	India	0.809	0.107	0.085	Q1
PI288347	India	0.144	0.027	0.829	Q3
PI288355	India	0.474	0.348	0.178	Q1Q2Q3
PI288359	India	0.356	0.431	0.212	Q1Q2Q3
PI288365	India	0.644	0.19	0.166	Q1
PI288377	India	0.051	0.002	0.948	Q3
PI288386	India	0.133	0.108	0.759	Q3
PI288390	India	0	0	1	Q3
PI288394	India	0	0	0.999	Q3
PI288413	India	0.402	0.424	0.175	Q1Q2Q3
PI288415	India	0.263	0.38	0.356	Q1Q2Q3
PI288424	India	0.39	0.283	0.327	Q1Q2Q3
PI288427	India	0.466	0.169	0.365	Q1Q2Q3
PI288432	India	0.753	0.101	0.146	Q1
PI288442	India	0.969	0.024	0.006	Q1
PI288737	India	0.054	0.945	0	Q2
PI288739	India	0.045	0.954	0.001	Q2
PI288741	India	0.043	0.956	0.001	Q2
PI288745	India	0.003	0.997	0	Q2

PI288751	India	0.246	0.753	0.001	Q2
PI288756	India	0.277	0.72	0.002	Q2
PI288762	India	0.003	0.458	0.54	Q1Q2Q3
PI322844	India	0.677	0.048	0.274	Q1
PI323083	India	0.955	0.044	0.001	Q1
PI338813	India	0.99	0.01	0	Q1
PI338872	India	0.998	0	0.001	Q1
PI338878	India	0.616	0.236	0.149	Q1
PI338884	India	0.937	0.062	0.001	Q1
PI338891	India	0.998	0.002	0	Q1
PI340224	India	0.921	0.004	0.075	Q1
PI340227	India	0.958	0.035	0.007	Q1
PI340235	India	0.967	0.001	0.032	Q1
PI340237	India	0.983	0.002	0.015	Q1
PI340240	India	0.876	0.003	0.121	Q1
PI340243	India	0.821	0.001	0.179	Q1
PI340246	India	0.837	0.029	0.134	Q1
PI340255	India	0.762	0.087	0.152	Q1
PI275323	India	0.575	0.002	0.423	Q1
PI288408	India	0	0	0.999	Q3
PI288425	India	0.61	0.387	0.003	Q1
PI288750	India	0.528	0.385	0.087	Q1Q2Q3
PI288758	India	0.157	0.788	0.056	Q2
PI322775	India	0.441	0.551	0.008	Q2
PI338796	India	0.986	0.001	0.013	Q1
PI338863	India	0.991	0.008	0.001	Q1
PI338870	India	0.941	0.02	0.038	Q1
PI340515	India	0.76	0.119	0.121	Q1
PI426635	Pakistan	0.84	0.033	0.128	Q1
PI164592	India	0.771	0.036	0.193	Q1
PI250212	Pakistan	0.84	0.033	0.127	Q1
PI271542	India	0.901	0.083	0.016	Q1
PI271545	India	0.964	0.035	0.001	Q1
PI271549	India	0.5	0.05	0.45	Q1Q2Q3
PI288418	India	0.399	0.316	0.285	Q1Q2Q3
PI288744	India	0.023	0.976	0.001	Q2
PI288749	India	0.036	0.942	0.022	Q2
PI288757	India	0.055	0.939	0.007	Q2
PI340511	India	0.888	0.018	0.094	Q1
PI340516	India	0.799	0.112	0.089	Q1
PI593054	USA	0.786	0.054	0.16	Q1
PI593059	USA	0.784	0.036	0.181	Q1
PI688679	USA	0.838	0.006	0.157	Q1
PI158118	India	0.99	0.004	0.006	Q1
PI158121	India	0.975	0.023	0.001	Q1
PI158124	India	0.91	0.077	0.013	Q1
PI158129	India	0.945	0.023	0.032	Q1
PI163104	India	0.915	0.003	0.082	Q1
PI164353	India	0.977	0.013	0.01	Q1

PI164476	India	0.79	0.008	0.202	Q1
PI164486	India	0.752	0.001	0.247	Q1
PI164801	India	0.934	0.002	0.064	Q1
PI176373	India	0.931	0.067	0.001	Q1
PI176378	India	0.878	0.034	0.088	Q1
PI179685	India	0.652	0.05	0.298	Q1
PI179926	India	0.964	0.035	0.001	Q1
PI179929	India	0.982	0.007	0.011	Q1
PI180288	India	0.793	0.061	0.146	Q1
PI180434	India	0.486	0.274	0.24	Q1Q2Q3
PI182969	India	0.483	0.494	0.023	Q1Q2Q3
PI198296	India	0.609	0.109	0.283	Q1
PI212987	India	0.698	0.004	0.298	Q1
PI214041	India	0.849	0.056	0.095	Q1
PI215590	India	0.999	0	0	Q1
PI217923	India	0.816	0.116	0.068	Q1
PI217925	India	0.736	0	0.264	Q1
PI236478	India	0.532	0.079	0.389	Q1Q2Q3
PI250213	Pakistan	0.963	0.018	0.019	Q1
PI250357	Pakistan	0.998	0.001	0.001	Q1
PI250359	Pakistan	0.956	0.002	0.042	Q1
PI253182	USA	0.954	0.001	0.045	Q1
PI253185	USA	0.876	0.001	0.123	Q1
PI253187	USA	0.804	0.02	0.176	Q1
PI254368	India	0.383	0.007	0.609	Q3
PI262151	Pakistan	0.945	0.001	0.054	Q1
PI262153	Pakistan	0.846	0.11	0.044	Q1
PI262156	Pakistan	0.964	0.032	0.004	Q1
PI263406	USA	0.471	0.215	0.314	Q1Q2Q3
PI263875	India	0.731	0.053	0.216	Q1
PI263877	India	0.763	0.158	0.079	Q1
PI263880	India	0.558	0.092	0.35	Q1
PI263882	India	0.455	0.253	0.293	Q1Q2Q3
PI263884	India	0.429	0.115	0.456	Q1Q2Q3
PI263886	India	0.418	0.133	0.449	Q1Q2Q3
PI263890	India	0.906	0.011	0.083	Q1
PI263892	India	0.921	0.043	0.036	Q1
PI263894	India	0.825	0.082	0.093	Q1
PI263896	India	0.926	0.001	0.074	Q1
PI263898	India	0.712	0.125	0.163	Q1
PI263900	India	0.602	0.038	0.361	Q1
PI268228	USA	0.743	0.178	0.079	Q1
PI271534	India	0.997	0.001	0.002	Q1
PI271540	India	0.778	0.103	0.12	Q1
PI271546	India	0.894	0.089	0.017	Q1
PI271551	India	0.494	0.059	0.447	Q1Q2Q3
PI288351	India	0.159	0.198	0.643	Q3
PI288357	India	0.493	0.304	0.203	Q1Q2Q3
PI288362	India	0.575	0.249	0.176	Q1

PI288369	India	0.117	0.218	0.665	Q3
PI288385	India	0.136	0.109	0.754	Q3
PI288388	India	0.029	0.004	0.966	Q3
PI288392	India	0	0	1	Q3
PI288395	India	0.19	0.179	0.631	Q3
PI288414	India	0.349	0.342	0.309	Q1Q2Q3
PI288419	India	0.245	0.419	0.336	Q1Q2Q3
PI288426	India	0.536	0.161	0.303	Q1Q2Q3
PI288429	India	0.298	0.276	0.425	Q1Q2Q3
PI288435	India	0.961	0.003	0.035	Q1
PI288443	India	0.518	0.48	0.001	Q1Q2Q3
PI288738	India	0.056	0.926	0.018	Q2
PI288740	India	0	0.999	0	Q2
PI288742	India	0.024	0.972	0.005	Q2
PI288747	India	0.257	0.73	0.012	Q2
PI288755	India	0.151	0.815	0.034	Q2
PI288760	India	0.078	0.917	0.005	Q2
PI288407	India	0.358	0.065	0.576	Q3
PI323045	India	0.719	0.077	0.204	Q1
PI338745	India	0.999	0	0.001	Q1
PI338847	India	0.997	0.001	0.002	Q1
PI338877	India	0.795	0.092	0.113	Q1
PI338883	India	0.797	0.165	0.038	Q1
PI338890	India	0.916	0.082	0.001	Q1
PI338896	India	0.825	0.043	0.132	Q1
PI340226	India	0.989	0.01	0.001	Q1
PI340228	India	0.952	0.003	0.044	Q1
PI340236	India	0.965	0.004	0.031	Q1
PI340239	India	0.819	0.007	0.174	Q1
PI340241	India	0.828	0.001	0.171	Q1
PI340244	India	0.82	0.013	0.166	Q1
PI340253	India	0.769	0.006	0.225	Q1

Note: Q refers to the group from population structure analysis.

Supplementary Table S2. Population structure matrix at K = 2.

Genotypes	Origin	Q1	Q2	Group
PI275322	India	0.919	0.081	Q1
PI288389	India	1	0	Q1
PI288409	India	0.999	0.001	Q1
PI288743	India	0.403	0.597	Q2
PI288754	India	0.378	0.622	Q2
PI288759	India	0.656	0.344	Q1
PI323002	India	0.506	0.494	Q1Q2
PI338811	India	1	0	Q1
PI338865	India	0.999	0.001	Q1
PI340513	India	0.822	0.178	Q1
PI426634	Pakistan	0.924	0.076	Q1
PI158119	India	0.959	0.041	Q1
PI164765	India	0.99	0.01	Q1
PI253184	USA	0.953	0.047	Q1
PI271544	India	0.991	0.009	Q1
PI271548	India	0.94	0.06	Q1
PI288384	India	0.749	0.251	Q1
PI288431	India	0.416	0.584	Q2
PI288748	India	0.02	0.98	Q2
PI288752	India	0.137	0.863	Q2
PI288761	India	0.525	0.475	Q1Q2
PI340514	India	0.925	0.075	Q1
PI593048	USA	0.926	0.074	Q1
PI593058	USA	0.965	0.035	Q1
PI671848	USA	0.992	0.008	Q1
PI116034	India	0.995	0.005	Q1
PI158120	India	0.944	0.056	Q1
PI158123	India	0.999	0.001	Q1
PI158126	India	0.883	0.117	Q1
PI163103	India	0.963	0.037	Q1
PI164170	India	0.999	0.001	Q1
PI164386	India	0.997	0.003	Q1
PI164477	India	0.998	0.002	Q1
PI164799	India	1	0	Q1
PI165511	India	0.97	0.03	Q1
PI176375	India	0.942	0.058	Q1
PI179682	India	0.953	0.047	Q1
PI179686	India	0.996	0.004	Q1
PI179928	India	0.996	0.004	Q1
PI180285	India	0.962	0.038	Q1
PI180433	India	0.98	0.02	Q1
PI182968	India	0.229	0.771	Q2
PI186477	India	0.914	0.086	Q1
PI198297	India	0.998	0.002	Q1
PI212988	India	0.995	0.005	Q1
PI214319	India	0.958	0.042	Q1

PI215591	India	0.999	0.001	Q1
PI217924	India	0.996	0.004	Q1
PI223685	India	0.996	0.004	Q1
PI236479	India	0.992	0.008	Q1
PI250214	Pakistan	0.999	0.001	Q1
PI250358	Pakistan	0.996	0.004	Q1
PI250360	Pakistan	1	0	Q1
PI253183	USA	0.999	0.001	Q1
PI253186	USA	1	0	Q1
PI254367	India	0.993	0.007	Q1
PI255928	India	0.995	0.005	Q1
PI262152	Pakistan	0.996	0.004	Q1
PI262154	Pakistan	0.896	0.104	Q1
PI262157	Pakistan	0.908	0.092	Q1
PI263874	India	0.91	0.09	Q1
PI263876	India	0.891	0.109	Q1
PI263879	India	0.808	0.192	Q1
PI263881	India	0.858	0.142	Q1
PI263883	India	0.853	0.147	Q1
PI263885	India	0.952	0.048	Q1
PI263888	India	0.897	0.103	Q1
PI263891	India	0.982	0.018	Q1
PI263893	India	0.967	0.033	Q1
PI263895	India	0.933	0.067	Q1
PI263897	India	0.977	0.023	Q1
PI263899	India	0.924	0.076	Q1
PI263901	India	0.811	0.189	Q1
PI268229	USA	0.999	0.001	Q1
PI271535	India	0.926	0.074	Q1
PI271541	India	0.952	0.048	Q1
PI271550	India	0.891	0.109	Q1
PI288347	India	0.996	0.004	Q1
PI288355	India	0.668	0.332	Q1
PI288359	India	0.59	0.41	Q1
PI288365	India	0.817	0.183	Q1
PI288377	India	1	0	Q1
PI288386	India	0.947	0.053	Q1
PI288390	India	1	0	Q1
PI288394	India	1	0	Q1
PI288413	India	0.589	0.411	Q1
PI288415	India	0.652	0.348	Q1
PI288424	India	0.744	0.256	Q1
PI288427	India	0.856	0.144	Q1
PI288432	India	0.902	0.098	Q1
PI288442	India	0.966	0.034	Q1
PI288737	India	0.055	0.945	Q2
PI288739	India	0.048	0.952	Q2
PI288741	India	0.043	0.957	Q2
PI288745	India	0.003	0.997	Q2

PI288751	India	0.244	0.756	Q2
PI288756	India	0.279	0.721	Q2
PI288762	India	0.601	0.399	Q1
PI322844	India	0.963	0.037	Q1
PI323083	India	0.946	0.054	Q1
PI338813	India	0.977	0.023	Q1
PI338872	India	1	0	Q1
PI338878	India	0.772	0.228	Q1
PI338884	India	0.923	0.077	Q1
PI338891	India	0.997	0.003	Q1
PI340224	India	0.999	0.001	Q1
PI340227	India	0.946	0.054	Q1
PI340235	India	0.999	0.001	Q1
PI340237	India	0.999	0.001	Q1
PI340240	India	0.998	0.002	Q1
PI340243	India	1	0	Q1
PI340246	India	0.978	0.022	Q1
PI340255	India	0.918	0.082	Q1
PI275323	India	1	0	Q1
PI288408	India	1	0	Q1
PI288425	India	0.602	0.398	Q1
PI288750	India	0.623	0.377	Q1
PI288758	India	0.22	0.78	Q2
PI322775	India	0.446	0.554	Q2
PI338796	India	1	0	Q1
PI338863	India	0.993	0.007	Q1
PI338870	India	0.98	0.02	Q1
PI340515	India	0.884	0.116	Q1
PI426635	Pakistan	0.97	0.03	Q1
PI164592	India	0.969	0.031	Q1
PI250212	Pakistan	0.971	0.029	Q1
PI271542	India	0.914	0.086	Q1
PI271545	India	0.946	0.054	Q1
PI271549	India	0.969	0.031	Q1
PI288418	India	0.71	0.29	Q1
PI288744	India	0.025	0.975	Q2
PI288749	India	0.062	0.938	Q2
PI288757	India	0.065	0.935	Q2
PI340511	India	0.989	0.011	Q1
PI340516	India	0.888	0.112	Q1
PI593054	USA	0.951	0.049	Q1
PI593059	USA	0.971	0.029	Q1
PI688679	USA	0.996	0.004	Q1
PI158118	India	0.992	0.008	Q1
PI158121	India	0.967	0.033	Q1
PI158124	India	0.921	0.079	Q1
PI158129	India	0.987	0.013	Q1
PI163104	India	0.998	0.002	Q1
PI164353	India	0.985	0.015	Q1

PI164476	India	0.996	0.004	Q1
PI164486	India	1	0	Q1
PI164801	India	0.999	0.001	Q1
PI176373	India	0.929	0.071	Q1
PI176378	India	0.968	0.032	Q1
PI179685	India	0.962	0.038	Q1
PI179926	India	0.963	0.037	Q1
PI179929	India	0.994	0.006	Q1
PI180288	India	0.942	0.058	Q1
PI180434	India	0.742	0.258	Q1
PI182969	India	0.504	0.496	Q1Q2
PI198296	India	0.901	0.099	Q1
PI212987	India	0.998	0.002	Q1
PI214041	India	0.944	0.056	Q1
PI215590	India	0.999	0.001	Q1
PI217923	India	0.883	0.117	Q1
PI217925	India	1	0	Q1
PI236478	India	0.942	0.058	Q1
PI250213	Pakistan	0.984	0.016	Q1
PI250357	Pakistan	1	0	Q1
PI250359	Pakistan	0.997	0.003	Q1
PI253182	USA	1	0	Q1
PI253185	USA	1	0	Q1
PI253187	USA	0.986	0.014	Q1
PI254368	India	0.999	0.001	Q1
PI262151	Pakistan	0.998	0.002	Q1
PI262153	Pakistan	0.888	0.112	Q1
PI262156	Pakistan	0.967	0.033	Q1
PI263406	USA	0.806	0.194	Q1
PI263875	India	0.955	0.045	Q1
PI263877	India	0.841	0.159	Q1
PI263880	India	0.924	0.076	Q1
PI263882	India	0.767	0.233	Q1
PI263884	India	0.907	0.093	Q1
PI263886	India	0.889	0.111	Q1
PI263890	India	0.987	0.013	Q1
PI263892	India	0.95	0.05	Q1
PI263894	India	0.916	0.084	Q1
PI263896	India	0.999	0.001	Q1
PI263898	India	0.88	0.12	Q1
PI263900	India	0.976	0.024	Q1
PI268228	USA	0.822	0.178	Q1
PI271534	India	0.999	0.001	Q1
PI271540	India	0.898	0.102	Q1
PI271546	India	0.903	0.097	Q1
PI271551	India	0.965	0.035	Q1
PI288351	India	0.856	0.144	Q1
PI288357	India	0.712	0.288	Q1
PI288362	India	0.76	0.24	Q1

PI288369	India	0.847	0.153	Q1
PI288385	India	0.943	0.057	Q1
PI288388	India	1	0	Q1
PI288392	India	1	0	Q1
PI288395	India	0.88	0.12	Q1
PI288414	India	0.682	0.318	Q1
PI288419	India	0.615	0.385	Q1
PI288426	India	0.858	0.142	Q1
PI288429	India	0.761	0.239	Q1
PI288435	India	0.993	0.007	Q1
PI288443	India	0.514	0.486	Q1Q2
PI288738	India	0.078	0.922	Q2
PI288740	India	0.001	0.999	Q2
PI288742	India	0.031	0.969	Q2
PI288747	India	0.269	0.731	Q2
PI288755	India	0.192	0.808	Q2
PI288760	India	0.086	0.914	Q2
PI288407	India	0.955	0.045	Q1
PI323045	India	0.93	0.07	Q1
PI338745	India	1	0	Q1
PI338847	India	0.999	0.001	Q1
PI338877	India	0.909	0.091	Q1
PI338883	India	0.828	0.172	Q1
PI338890	India	0.91	0.09	Q1
PI338896	India	0.96	0.04	Q1
PI340226	India	0.992	0.008	Q1
PI340228	India	0.993	0.007	Q1
PI340236	India	0.996	0.004	Q1
PI340239	India	0.996	0.004	Q1
PI340241	India	0.999	0.001	Q1
PI340244	India	0.991	0.009	Q1
PI340253	India	0.998	0.002	Q1

Note: Q refers to the group from population structure analysis.
